# Dynamic networks differentiate the language ability of children with cochlear implants

**DOI:** 10.3389/fnins.2023.1141886

**Published:** 2023-06-20

**Authors:** Nabin Koirala, Mickael L. D. Deroche, Jace Wolfe, Sara Neumann, Alexander G. Bien, Derek Doan, Michael Goldbeck, Muthuraman Muthuraman, Vincent L. Gracco

**Affiliations:** ^1^Child Study Center, Yale School of Medicine, Yale University, New Haven, CT, United States; ^2^Department of Psychology, Concordia University, Montreal, QC, Canada; ^3^Hearts for Hearing Foundation, Oklahoma City, OK, United States; ^4^Department of Otolaryngology – Head and Neck Surgery, University of Oklahoma Medical Center, Oklahoma City, OK, United States; ^5^University of Oklahoma College of Medicine, Oklahoma City, OK, United States; ^6^Department of Neurology, Neural Engineering with Signal Analytics and Artificial Intelligence (NESA-AI), Universitätsklinikum Würzburg, Würzburg, Germany; ^7^School of Communication Sciences and Disorders, McGill University, Montreal, QC, Canada

**Keywords:** cochlear implant, electroencephalography (EEG), language and reading, age of intervention, electrical source imaging (ESI)

## Abstract

**Background:**

Cochlear implantation (CI) in prelingually deafened children has been shown to be an effective intervention for developing language and reading skill. However, there is a substantial proportion of the children receiving CI who struggle with language and reading. The current study–one of the first to implement electrical source imaging in CI population was designed to identify the neural underpinnings in two groups of CI children with good and poor language and reading skill.

**Methods:**

Data using high density electroencephalography (EEG) under a resting state condition was obtained from 75 children, 50 with CIs having good (HL) or poor language skills (LL) and 25 normal hearing (NH) children. We identified coherent sources using dynamic imaging of coherent sources (DICS) and their effective connectivity computing time-frequency causality estimation based on temporal partial directed coherence (TPDC) in the two CI groups compared to a cohort of age and gender matched NH children.

**Findings:**

Sources with higher coherence amplitude were observed in three frequency bands (alpha, beta and gamma) for the CI groups when compared to normal hearing children. The two groups of CI children with good (HL) and poor (LL) language ability exhibited not only different cortical and subcortical source profiles but also distinct effective connectivity between them. Additionally, a support vector machine (SVM) algorithm using these sources and their connectivity patterns for each CI group across the three frequency bands was able to predict the language and reading scores with high accuracy.

**Interpretation:**

Increased coherence in the CI groups suggest overall that the oscillatory activity in some brain areas become more strongly coupled compared to the NH group. Moreover, the different sources and their connectivity patterns and their association to language and reading skill in both groups, suggest a compensatory adaptation that either facilitated or impeded language and reading development. The neural differences in the two groups of CI children may reflect potential biomarkers for predicting outcome success in CI children.

## Introduction

It is well known that early sensory loss creates the backdrop for changes in cortical and subcortical brain organization (Merabet and Pascual-Leone, 2010). For early deafness, there is a decrease in synaptic plasticity that results in morphological changes in children with hearing loss suggesting that early sensory deprivation creates a developmental delay in the myelination of the auditory neural pathway (Chang et al., 2012; Huang et al., 2015; Wu et al., 2020). The resulting auditory deprivation significantly impacts bilateral superior temporal gyri and the white matter fibers comprising the inferior fronto-occipital fasciculus, the superior longitudinal fasciculus, and the subcortical auditory pathway (Simon et al., 2020). Morphological structure of the auditory pathway in children diagnosed with congenital sensorineural hearing loss exhibit changes (reduced fractional anisotropy) in all portions of the auditory pathway (Feng et al., 2018; Wang et al., 2019). These differences have functional consequences for post-cochlear implantation success. For example, fractional anisotropy values obtained pre-surgery have been found to be negatively correlated with Categories of Auditory Performance (CAP) assessed at 12 months post implantation (Huang et al., 2015). Similarly, cortical thickness obtained pre surgery has been shown to predict variability in speech perception abilities 6 months post-surgery (Feng et al., 2018). At a network level, children with bilateral profound sensorineural hearing loss demonstrate changes in network connectivity along with reduced low frequency amplitude fluctuations in auditory, language and executive function brain areas and increased low frequency fluctuations in visual processing areas (Xia et al., 2017; Wang et al., 2021). However, after hearing restoration from a cochlear implant, network level changes have been rarely studied. Investigations using electroencephalography (EEG) (McGuire et al., 2021; Paul et al., 2021), functional near-infrared spectroscopy (fNIRS) (Pollonini et al., 2014; McKay et al., 2016; Olds et al., 2016; van de Rijt et al., 2016; Zhou et al., 2018), or even positron emission tomography (PET) (Giraud et al., 2000, 2001; Rouger et al., 2012; Petersen et al., 2013) have reported speech and language processing differences in adult users of CIs, including evidence for cross-modal plasticity (Doucet et al., 2006; Sandmann et al., 2012; Stropahl et al., 2015; Kim et al., 2016; Anderson et al., 2017; Chen et al., 2017a,b). However, there are only a handful of similar studies for children with CIs (Sharma et al., 2007; Sevy et al., 2010; Bortfeld, 2019; Pierotti et al., 2022) and currently no data on the more global organization of different networks.

In the last decade, it has been well evidenced that for children receiving cochlear implants, better outcomes are associated with earlier age of implantation (Geers, 2004; Niparko, 2004; Niparko et al., 2010; Bruijnzeel et al., 2016). Children implanted younger than 12 months demonstrate superior communication outcomes compared to children receiving CI at later ages (Geers et al., 2003; Leigh et al., 2013; Dettman et al., 2016). Similarly, better outcomes for children with CI were obtained when implanted before the age of 3.5 years (so called the sensitive period) (Sharma et al., 2005, 2007, 2009, 2015) with a suggestion of this sensitive period influencing different aspects of communication (Cartocci et al., 2021). While all of these outcomes are encouraging, there are a few caveats. In all standardized assessments, significant variability can be seen for the post-implantation communication outcomes (Niparko et al., 2010; Dettman et al., 2016; Yoshinaga-Itano et al., 2018). For example, from a large cohort implanted at <12 months of age, receptive vocabulary scores were within the normal range for 81% of the children (Dettman et al., 2016). In the same group their fundamental language skills were only within the normal range for 58% of the children. A longitudinal study of language development in a small cohort (*N* = 21) of early implanted children (5–18 months at implantation) in Norway found that in the 4 years post bilateral implantation, the expressive and receptive language gap in children with CIs gradually closed compared with NH children (Wie et al., 2020). However, at a later stage (6 years post implantation), expressive grammar skills were lower in the children with CIs and a gap in receptive vocabulary appeared and grew from 4 to 6 years post implantation. Literacy evaluations also reflect variable outcomes. A meta-analysis of 47 articles that examined reading skills of children with CIs found that phonological awareness, vocabulary, decoding and reading comprehension all were significantly lower than their hearing peers (Wang et al., 2021). Importantly, the magnitude of difference in emergent and later reading skill did not relate to age at testing, age at onset of deafness, age at implantation and implant use duration. Socio-economic and environmental factors such as family support and availability of early childhood resources have been shown to contribute (Chang et al., 2010; Silva et al., 2020). However, these factors alone are unable to explain the variability in the long-term literacy outcomes for children with CIs. Overall, there are unaccounted factors that impact successful language outcomes highlighting the continual challenges faced by children with CIs.

The functional consequences associated with neuroplastic crossmodal changes after sensory deprivation remain unclear. While the dominant perspective is that reorganization compensates for the sensory loss, results thus far do not unequivocally indicate that sensory deprivation results in markedly enhanced abilities in other senses (Singh et al., 2018). Rather than conferring functional benefits, the changes may result from minimizing undesirable physiological consequences of sensory loss (Dancause, 2006; Gagnon et al., 2013). For the children with CIs, the specifics of the cortical reorganization would have a huge impact in their post-implantation cognitive outcome. Hence understanding such changes can provide insight into the variable effects of hearing restoration during the early stage of development. The current study is one of the first to use non-invasive electrical source imaging (ESI) to assess differences in neural sources and their connectivity patterns at rest and relate them to differences in language and reading skill levels in children with CIs. ESI is an established evidenced-based non-invasive technique to detect and localize the cortical and subcortical signals (neuronal activity) recorded from scalp electrodes (Muthuraman et al., 2018; Michel and Brunet, 2019; Seeber et al., 2019). This method allows the reconstruction of neuronal activity in specific brain areas with millisecond resolution which is significantly superior to functional Magnetic Resonance Imaging (fMRI), enabling the measurement of real-time fluctuations in temporal dynamics of whole-brain neuronal networks (Michel and Murray, 2012). The technique has been widely used for quantifying neural activity in various neurological, and neuropsychological disorders and understanding neurocognitive functions (Michel et al., 2001). Some examples include the precise localization of the irritative zone and its impact in different brain networks in patients with focal epilepsy (Brodbeck et al., 2011; Megevand et al., 2014; Coito et al., 2016), obtaining the tremor sources in essential tremor and Parkinson’s disease patients (Muthuraman et al., 2018), detecting spontaneous brain activity (Coito et al., 2019), tracking cortical activities and induced changes in hearing loss and cochlear implanted patients (Glick and Sharma, 2017; Song et al., 2018), characterizing the functional topological brain network changes of consciousness patients (Rizkallah et al., 2019) etc. Moreover, the technique is extremely useful for the cochlear implanted population as the post-surgical brain imaging is not possible using fMRI.

## Materials and methods

### Participants

**FIGURE 1:**
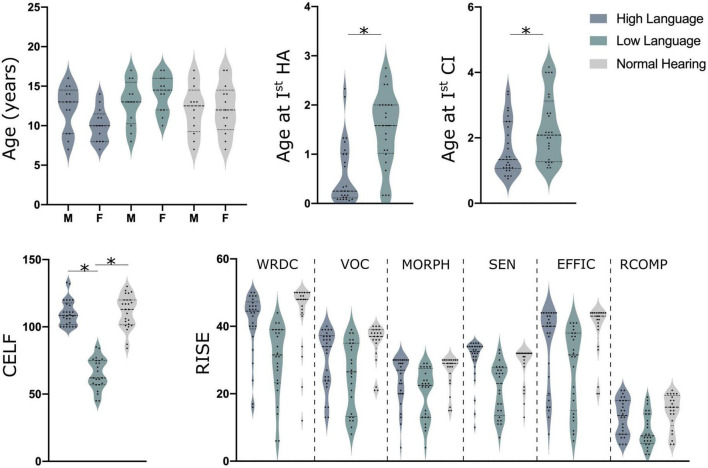
Demographics details along with CELF (clinical evaluation of language fundamentals) and RISE (reading inventory and scholastic evaluation) scores for all children. Here, HA indicates hearing aids (although none were used in this study), CI, cochlear implant; M, males and F indicates females, *indicates the significant difference of *p* < 0.05 for a *T*-Test.

Resting state electroencephalography (EEG) data was obtained from 75 children. Of the 75 children, 50 were children with congenital bilateral severe to profound hearing loss and implanted with cochlear implants and 25 were normal hearing controls [NH; mean age: 12.08 ± 3.1 years, see Figure 1 for details; additional details on demographics, therapy attendance and speech recognition performance is reported in Wolfe et al. (2021)]. A total of 45 children were bilaterally implanted with the second implantation occurring about 16–17 months after the first implant. The children with CIs were divided into two groups with significantly different language abilities based on their standard scores on the *Clinical Evaluation of Language Fundamentals – Fifth Edition* (CELF-5) (Wiig et al., 2013). CELF-5 scores are one of the most common tests to determine language aptitude in children with hearing loss (Geers et al., 2019). The children with good language outcome, hereafter High Language group (HL; *N* = 26; mean age: 11.5 ± 2.8 years) had a composite score of 100 or more on the CELF-5, whereas those with the poor language outcome post-implantation, hereafter Low Language group (LL; mean age: 13.9 ± 2.6 years) had a composite score of 85 or less on the CELF-5.

The following inclusion and exclusion criteria were implemented by a licensed speech–language pathologists for the selection of the children with CIs.

#### Inclusion criteria

At least one CI by 4 years of age.

Primary communication via listening and spoken language in American English (i.e., limited use of sign language in most daily listening settings).

Minimum of 6 h of CI use per day as indicated by data logging.

#### Exclusion criteria

No additional disabilities that could induce delays in language development.

No anatomical abnormalities that could cause delays in language development such as ossification after bacterial meningitis, cochlear nerve deficiency, or significant cochlear deformities.

### Data acquisition

Electroencephalography data for all participants was acquired using electrodes placed on the scalp with a 128-channel high density *Geodesic Sensor Net* (GSN) from Magstim Electrical geodesic Inc., Eugene, OR, USA. Resting state recording was carried out for 7 min (sampling frequency of 1024 Hz) in each participant while they were sitting in a comfortable chair watching *Inscapes*–a movie paradigm that features abstract shapes without a narrative or scene-cuts (Vanderwal et al., 2015). The impedance was kept under 10 kΩ throughout the measurement and the obtained data was analyzed offline. During the resting state acquisition, the cochlear implant sound processor was off, and the data was acquired in a quiet room with the background noise level measured at 35 dBA.

All participants completed a battery of audio and speech tests using the Pediatric Minimum Speech Test Battery (PMSTB) protocol (Uhler et al., 2017) and consonant– nucleus–consonant (CNC) test (Peterson and Lehiste, 1962) along with tests for language and reading skills. Speech recognition scores for the two CI groups were recently reported and differed substantially (Wolfe et al., 2021). CELF-5 was completed to determine language aptitude and the RISE (Reading Inventory and Scholastic Evaluation) (Sabatini et al., 2019) subtests for determining their reading ability were administered off-line. The RISE is an inventory testing six different components of reading skills including: Word Recognition and Decoding (WRDC), Vocabulary (VOC), Morphology (MORPH), Sentence Processing (SEN), Efficiency of Basic Reading Comprehension (EFFIC), and Reading Comprehension (RCOMP).

**FIGURE 2:**
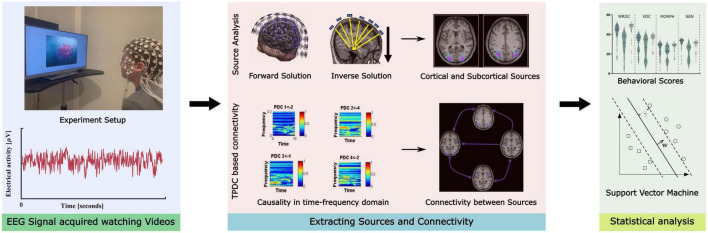
Illustration of the methodological pipeline implemented in the study.

All the experimental procedure was approved by the ethical committee board of the institution (Hearts for Hearing) and subjects’ written informed consent was obtained from their legal guardian. All study procedures were conducted in accordance with appropriate guidelines and following the norms of the Declaration of Helsinki. An illustration of the methodological pipeline is presented in Figure 2.

### Data analysis

#### Pre-processing and artifacts corrections

All the data was processed using MATLAB (Mathworks Inc., USA) based scripts. The EEG data was first segmented into numerous 1-s epochs, discarding all data segments with visible artifacts. Depending on the length (N) of the recording and the quality of the data, 380–400 1-s epochs (M) were used for analysis, such that *N* = LM, where *L* = 1024 points—based on sampling rate. The raw EEG data was low-pass filtered (fourth-order Butterworth filter; cut-off frequency: 300 Hz) to avoid aliasing, followed by high pass filtering at 0.5 Hz. A notch filter was used to filter out the 60, 120, and 180 Hz power line artifacts. The EEG data was then subjected to independent component analysis (ICA) (Kim et al., 2015) to remove the components representing the muscle artifacts, eye blinks, eye movements, and line noise. An average of 16 of the 128 components (16 ± 4.3, mean ± SD) were rejected, of which 4–6 were related to eye artifacts (4 ± 2.48), 2–4 were related to line noise (2 ± 1.46) and 4–6 were related to muscle artifacts (4 ± 3.56). The residual muscle artifacts were visually inspected, removed, and interpolated with the cubic interpolation method.

Electroencephalography data was then re-referenced to the common grand average reference of all EEG channels. Continuous data was first decomposed into time-frequency representation by using the multitaper method (Mitra and Pesaran, 1999; Muthuraman et al., 2010). In this method, explained in detail in Muthuraman et al. (2010), the spectrum is estimated by multiplying the data (*x(t)*) with *K* different windows (i.e., tapers) using a sliding time window for calculating the power spectrum (*S*_*MT*_)by discrete Fourier transformation as follows:


SM⁢T⁢(f)=1K⁢∑k=1K|X′k⁢(f)|2

Where, X′k⁢(f) is the Fourier transform of the windowed signal *x(t)*. For this analysis, the time step was 50 ms with overlapping windows of 1000 ms, providing an approximate time resolution of 50 ms and frequency resolution of 1 Hz. Moreover, in this study orthogonal tapers were used which have the advantage of minimizing spectral properties, along with the applied discrete prolate spheroidal sequences for overcoming undesirable effects such as local and broadband biases and uncertainty that increase the overall error variance (Slepian and Pollak, 1961; Percival and Walden, 1993). The power spectra across the electrodes were manually examined for quality control. Peaks in the spectrum were found in alpha, beta and gamma frequency bands for each group determined using an in-house algorithm. The algorithm works with overlaying three points in the power spectrum with the center point compared to the two neighboring points (on each side). The comparison is performed iteratively computing power differences between the three points and if the difference in the points on the side is less than two standard deviations, the center point is considered as a peak. If more than one peak is identified in a frequency band, then the highest peak is considered for analysis.

#### Source analysis

A brief description of this analysis is presented below, and more detail could be found in our previous publications (Muthuraman et al., 2008a,b, 2018). Dynamic imaging of coherent sources (DICS) (Sekihara and Scholz, 1996; Gross and Ioannides, 1999; Gross et al., 2001) was used to localize brain activity at specific frequency bands. To locate the origin (source) of specific EEG activity seen on the scalp, two sets of problems need to be solved commonly referred to as the forward and the inverse problems (Michel and Brunet, 2019).

The forward problem is the computation of the scalp potentials for a set of neural current sources which is usually solved by estimating the lead-field matrix with specified models for the brain (Wolters et al., 2007). For this study, the forward modeling was done using the surfaces of the compartments–scalp, skull, and brain, extracted from the standardized 3T-MRI age-based template (Sanchez et al., 2012) for each participant’s rounded-off age and the individual electrode locations. The reconstruction of the brain activity used the forward solution with a finite-element method (Wolters et al., 2007).

The inverse problem is finding the relation between the underlying neural activities and the electric potentials recorded on the scalp which is generally solved by a linear transformation using a spatial filter (van Veen et al., 2002). The spatial filter attenuates the signals from non-desired locations and allows signals generated only from a desired location in the brain for a given frequency band. A full description of the beamformer linear constrained minimum variance spatial filter is given elsewhere (van Veen et al., 1997; Muthuraman et al., 2018).

The output of the beamformer at a voxel in the brain can be defined as a weighted sum of the output of all EEG channels at the given location. The weights determine the spatial filtering characteristics of the beamformer and are selected to increase the sensitivity of signals from a voxel and reduce the contributions of signals from (noise) sources at different locations. The frequency components and their linear interactions are represented as a cross-spectral density matrix. In order to visualize power at a given frequency range, a linear transformation was used based on a constrained optimization problem that acts as a spatial filter (van Veen et al., 1997). The spatial filter assigned a specific value of power to each voxel. For a given source the beamformer weights for a location of interest are determined by the data covariance matrix and the lead-field matrix. A voxel size of 5 mm^3^ was used in this study, resulting in 3676 voxels covering the entire brain. Here, we analyzed the sources for Alpha (8–12 Hz), Beta (13–30 Hz), and Gamma (30–100 Hz) frequency range. The brain region peak voxels representing the strongest power in a specific frequency was identified for each individual participant. The peak power voxels were selected by a within-subject surrogate analysis to define the significance level, which was then used to identify areas in the brain as activated voxels for subsequent runs of the source analysis. Once brain region peak voxels were identified, their activity in source space was extracted from the surface EEG.

Further, all original source signals with several activated voxels were combined by estimating the second order spectra and computing a weighting scheme depending on the analyzed frequency range to form a pooled source signal estimate for each region as previously described (Rosenberg et al., 1989; Amjad et al., 1997). The pooled source signal estimated was then used as reference to find subsequent significant coherent sources at each frequency band separately. The individual maps of the strongest coherence were spatially normalized, averaged and registered on a standard Montreal Neurological Institute (MNI) template brain using statistical parametric mapping (SPM8) for visualization.

#### Connectivity analysis

Connectivity analysis was conducted using the time–frequency causality estimation method of temporal partial directed coherence (TPDC). TPDC is based on dual-extended Kalman filtering (Haykin, 2001; Wan and Nelson, 2001) and allows the estimation of time-dependent autoregressive coefficients. The time-frequency analysis focuses on a particular frequency and examines the dynamics of its causality at that frequency. In this method, one extended Kalman filter estimates the states and feeds this information to a subsequent Kalman filter that estimates the model parameters and shares this information with the previous estimate. Hence, by using two Kalman filters in parallel, we were able to estimate the states and model parameters of the system at each time instant. The causality at each instant were computed as the partial directed coherence using time-dependent multivariate autoregressive coefficients. The same three frequency bands used for source analysis were also used for this analysis.

After estimating the TPDC values, the statistical significance was calculated from the applied data using a bootstrapping algorithm detailed elsewhere (Kaminski et al., 2001; Muthuraman et al., 2018). In short, for each participant, the original time series was divided into smaller non-overlapping windows, and these windows were randomly shuffled to create a new time series. A multivariate autoregressive model was fitted to the shuffled time series, and TPDC was estimated. The iteration was performed for 1000 times, and the average TPDC value was taken as the significant threshold for all connections. An open source MATLAB package autoregressive fit (ARFIT) (Arnold and Tapio, 2001) was used for estimating the autoregressive coefficients from the spatially filtered source signals. As volume conduction severely limits the neurophysiological interpretability of sensor-space connectivity analyses (Gómez-Herrero et al., 2008; Schoffelen and Gross, 2009), we checked the reliability of our connectivity using time reversal technique (TRT) (Haufe et al., 2013). TRT was applied as a second significance test on the connections already identified by TPDC using bootstrapping algorithm as a data-driven surrogate significance test.

#### Prediction and statistical analysis

The total data lengths among the subjects were tested for similar lengths using a non-parametric Friedman test for independent samples (*n* = 75, *P* > 0.05). The statistical significance of the sources (*n* = 75, *P* = 0.01) was tested using a within-subject surrogate analysis (Kaminski et al., 2001). In this analysis, the time series data from each channel was shuffled randomly and independently to create a surrogate data set. A model is then fitted to this surrogate dataset to derive causal measures from the model. This process is carried out iteratively, each time performed in a new surrogate dataset to create an empirical distribution for the causal measures. Since the construction is designed to have no interaction among the channels, these distributions give the estimator behavior for the null hypothesis case (Kaminski et al., 2001). A Monte-Carlo test of 1000 random permutations was carried out, and the *p*-value at each iteration was calculated (Maris and Oostenveld, 2007; Maris et al., 2007). We calculated the 99% confidence limit, and values of coherence below this confidence limit were considered to lack linear dependence between the two source signals. Hence, the 99th percentile *p*-value was taken as statistically significant for each subject (Moeller et al., 2013).

To investigate the significance of these source coherence and connectivity values, we used a support vector machine (SVM) algorithm to predict the behavioral (CELF and RISE) scores for high language ability CI (HL) and low language ability CI (LL) groups. For the model, the predictors were either the obtained coherence magnitude value of the sources or TPDC-based connectivity values between those sources from all frequency bands and the standard scores of CELF and sub-tests of RISE. SVM is a powerful tool for non-linear classification between two data sets which looks for an optimally separating threshold between the two data sets by maximizing the margin between classes’ closest points (Cortes and Vapnik, 1995). Here, we used the polynomial function kernel for this projection due to its good performance as discussed in Cortes and Vapnik (1995) and use the grid search (min = 1; max = 10) to find the few optimal input parameters and gamma (0.25). The selection was checked by fivefold cross validation by taking 75% of the data for training and 25% for testing.

#### Data sharing statement

Identifiable patients’ data used for the study cannot be shared because of the agreement signed with the participants. However, partially analyzed, deidentified electrophysiological and behavioral data could be shared with appropriate request and IRB approval letter to the corresponding author. The relevant codes used in the study can be obtained with a request to corresponding author.

## Results

**FIGURE 3:**
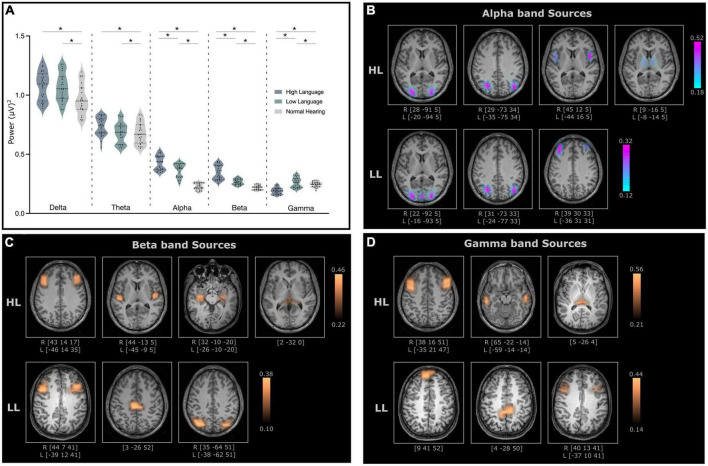
**(A)** Relative power for all frequency bands for high language (HL) and low language (LL) cochlear implanted children when compared to normal hearing (NH) cohort. The significant difference (*p*-values < 0.05) between the groups is indicated with an asterisk. **(B)** Cortical and subcortical sources of alpha band activity **(C)** beta band activity and **(D)** gamma band activity with significantly higher coherence in the HL and LL groups when compared to the NH group. Here, the coordinates below each source indicates the MNI coordinates of the location with the highest coherence source amplitude.

Significant differences (ANCOVA with age and sex as covariates, Bonferroni’s corrected, *p* < 0.05) in absolute power (representing amount of neural activity in certain frequency bands of the signal) were observed between normal hearing and CI groups for all bands [Delta: *F*_(2, 70)_ = 6.99, *p* = 0.002, Theta: *F*_(2, 70)_ = 6.99, *p* = 0.021, Alpha: *F*_(2, 70)_ = 97.92, *p* < 0.001, Beta: *F*_(2, 70)_ = 66.53, *p* < 0.001, Gamma: *F*_(2, 70)_ = 28.49, *p* < 0.001]. However, the difference between the two CI groups were only observed for the alpha, beta and gamma bands (Figure 3A). Because the focus was on identifying neural differences in cochlear implanted children with good and poor literacy outcomes, the source localization was only conducted for the three bands that differed for the HL and LL groups.

### Sources

Electroencephalography source localization analysis detected cortical and subcortical sources with higher coherence in the HL and LL–CI groups when compared to the NH group. All identified sources were statistically significant (*P* < 0.005 for within subject surrogate analyses) based on a Monte Carlo random permutation test across all subjects per group. The mean coherence values (representing total interaction strength between sources) were significantly stronger (*P* < 0.05) in the HL group compared to the LL group when compared to NH for all frequencies (Alpha: HL: 0.333 ± 0.11, LL: 0.216 ± 0.06; Beta: HL: 0.334 ± 0.07, LL: 0.252 ± 0.08; Gamma: HL: 0.386 ± 0.10, LL: 0.270 ± 0.09). Moreover, these sources with significantly greater coherence amplitude also differed in the locations for the HL and LL groups.

### Alpha band sources

The identified alpha band sources for the two CI groups relative to the NH group are presented in Figure 3B. Four sources were identified for the HL group (lingual gyrus, angular gyrus, Broca’s area, thalamus–mediodorsal nucleus), and three sources were identified for the LL group (lingual gyrus, angular gyrus, mid frontal gyrus). Interestingly, two of the sources for the HL that were not present for the LL group were in Broca’s area and the thalamus.

### Beta band sources

For the most part, the beta band sources for the two groups (four for the HL and three for the LL groups) differed. Sources for the HL group were located in mid frontal/Broca’s area, the insula/Heschl’s gyrus, the hippocampus and the thalamus (pulvinar). Sources for the LL group were located bilaterally in the mid frontal gyrus, the paracentral lobule and the angular gyrus (Figure 3C).

### Gamma band sources

Gamma band sources for the two groups also differed with the sources from the HL group located in the mid frontal gyrus, the middle temporal gyrus and the pulvinar. The sources from the LL group were located in the superior frontal gyrus, the paracentral lobule/posterior cingulate and the mid frontal gyrus (Figure 3D).

A summary of the identified source locations for the two groups is presented in Table 1.

**TABLE 1:** Summary of all identified sources (listed in decreasing strength) for both high language and low language group with the corresponding brain regions based on the Brodmann atlas.

High language		Low language	
**Sources**	**ROI**	**Sources**	**ROI**
Alpha–S1	BA 17 (visual cortex)	Alpha–S1	BA 17 (visual cortex)
Alpha–S2	BA 39 (angular gyrus)	Alpha–S2	BA 39 (angular gyrus)
Alpha–S3	BA 44 (precentral gyrus)	Alpha–S3	BA 9 (mid frontal gyrus)
Alpha–S4	Thalamus		
Beta–S1	BA 8/44 (mid frontal/operculum)	Beta–S1	BA 8 (frontal eye fields)
Beta–S2	BA 13 (insula/Heschl’s)	Beta–S2	BA 6/31 (SMA/dorsal PCC)
Beta–S3	Hippocampus	Beta–S3	BA 39 (angular gyrus)
Beta–S4	Thalamus		
Gamma–S1	BA 6 (mid frontal gyrus)	Gamma–S1	Superior frontal gyrus
Gamma–S2	BA 21 (middle temporal gyrus)	Gamma–S2	BA 31 (paracentral/posterior cingulate)
Gamma–S3	Thalamus	Gamma–S3	BA 6 (mid frontal gyrus)

The name of each region was based on the location where the highest coherence source amplitude was found. The MNI coordinates of these locations could be found in Figures 1–3 for each frequency band, respectively.

### Connectivity

**FIGURE 4:**
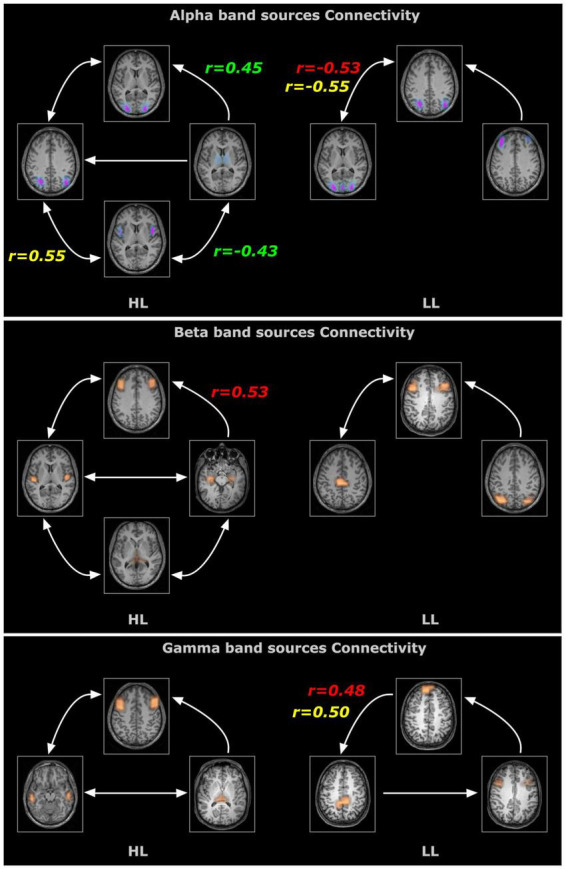
Time resolved partial directed coherence (TPDC)–based directional connectivity analysis showing the effective connectivity between the sources of alpha, beta and gamma band activity with the direction of information flow for the HL and LL groups. Here, the *r*-values indicate the correlations between the TPDC based connectivity to the timing of hearing restoration intervention [Age of first CI (in red), age of bilateral CI (in yellow), age of hearing aid fitting (in green)] as detailed in Table 2.

Effective connectivity analysis was used to assess the strength and directionality of the connections among the sources indicating the neural information flow. For the obtained sources, all connections (except two) survived the surrogate significant testing (Figure 4). The connectivity pattern between two groups differed in terms of sources involved, number of connections and the directionality of the connectivity for all three frequency bands. For Alpha band sources, the HL group contained all bidirectional connections except for those from the thalamus but for LL group, the bidirectional connection only appeared between visual cortex and angular gyrus. The connectivity analysis of the Beta band sources also revealed differential connectivity patterns between the HL and LL group with the HL group predominantly bidirectional except for the connection from hippocampus to the middle frontal gyrus. For Gamma band, all connectivity for LL group were unidirectional however for the HL group all except one connection were bidirectional with the pulvinar and mid frontal sources bidirectionally coupled to the temporal cortex.

### Prediction of language and reading measures

**FIGURE 5:**
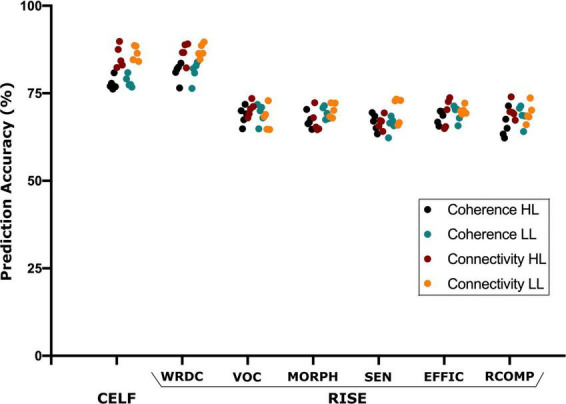
Support vector machine (SVM) based prediction accuracy using coherence and TPDC–based connectivity values for CELF (clinical evaluation of language fundamentals) and RISE (reading inventory and scholastic evaluation) sub-scores. Here, the SVM could predict all language and reading test scores however CELF and RISE-subtest–word recognition and decoding (WRDC) obtained the highest accuracy among them. Here: other subtests of RISE includes VOC, vocabulary; MORPH, morphology; SEN, sentence processing; EFFIC, efficiency of basic reading comprehension; RCOMP, reading comprehension.

The SVM analysis was able to predict the language (CELF) and reading (RISE) scores using the source and connectivity measures in both the HL and LL groups. Using source coherence amplitude and the connectivity values between the sources as predictors for each group the SVM analysis was able to predict the CELF scores of each group with predictive accuracy higher than 85%. Similarly, for the RISE scores, SVM’s prediction accuracy reached up to 88% for the word recognition (WDRC) scores with other sub-test accuracies ranging between 68 and 75% (Figure 5).

### Coherence and connectivity with timing of hearing interventions

In order to evaluate whether these differences observed under resting state conditions were associated with the timing and kind of hearing restoration differences in the groups, we further examined the relationship of age of first CI, age of bilateral CI and age of hearing aid fitting to the coherence sources and their connectivity. Table 2 lists the significant results for each of those variables for the groups. For the HL group, age of first CI was positively correlated with the magnitude of the coherence for the Beta band source around Broca’s area (BA 8/44) (*r* = 0.4121, *p* = 0.0364) and age of bilateral CI was negatively correlated with the insula/Heschl’s region (BA 13, BA 41) (*r* = −0.4698, *p* = 0.0154). None of the sources for the LL group were related to the timing of hearing restoration. Connectivity values between the sources were also associated with age of hearing aid fitting and age of first CI for the HL group and with age of bilateral CI for both groups. For the HL group, age of hearing aid fitting was related to two Alpha band connectivity patterns—the precentral gyrus (BA 44) to the thalamus (medial dorsal) (*r* = −0.4306, *p* = 0.0281) and the thalamus to the visual cortex (BA 17) (*r* = 0.4446, *p* = 0.02285). Further, age of first CI was related to Beta band connectivity between the hippocampus and the thalamus (pulvinar) (*r* = 0.5314, *p* = 0.0052) and for Alpha band connectivity between the visual cortex (BA 17) and the precentral gyrus (BA 44) for age of bilateral CI (*r* = 0.5507, *p* = 0.0035). For the LL group, age of bilateral CI was related to Alpha band connectivity between the angular gyrus (BA 39) and the visual cortex (BA 17) (*r* = −0.5573, *p* = 0.0106) and for Gamma band connectivity between the superior frontal gyrus and the paracentral lobe/posterior cingulate (BA 31) (*r* = 0.4983, *p* = 0.0253). All significant associations are indicated in the connectivity map in Figure 5.

**TABLE 2A:** Relationship between the coherence amplitude of the sources and the timing of hearing restoration intervention (Age of first CI, age of bilateral CI).

High language			Low language		
**Coherence (source analysis)**					
**Source**	**Correlation**	**Frequency range**	**Source**	**Correlation**	**Frequency range**
**Age of first CI**					
MFG/BA 44	*r* = 0.41	Beta			
**Age of Bilateral CI**					
Insula/Heschl’s	*r* = −0.47	Beta			

MFG, mid frontal gyrus; BA 44, Brodmann area 44-Broca’s area.

**TABLE 2B:** Relationship between the TPDC connectivity between the sources to the timing of hearing restoration intervention (Age of first CI, age of bilateral CI, age of hearing aid fitting).

High language			Low language		
**Connectivity (TPDC analysis)**					
**Regions**	**Correlation**	**Frequency range**	**Regions**	**Correlation**	**Frequency range**
**Age of first CI**					
Hipp → Thalamus	*r* = 0.53	Beta	BA 39 → BA 17	*r* = −0.53	Alpha
			SFG → PCL/PCG	*r* = 0.48	Gamma
**Age of Bilateral CI**					
BA 17 → BA 44	*r* = 0.55	Alpha	BA 39 → BA 17	*r* = −0.55	Alpha
			SFG → PCL/PCG	r = 0.50	Gamma
**Age of hearing aid fitting**					
BA 44 → Thalamus	*r* = −0.43	Alpha			
Thalamus BA → 17	*r* = 0.45	Alpha			

Hipp, Hippocampus; BA 44, Brodmann area 44-Broca’s area; SFG, superior frontal gyrus; PCL, paracentral lobule; PCG, posterior cingulate gyrus; BA 17, Brodmann area 17–visual cortex; BA 39, Brodmann area 39—angular gyrus. The connectivity correlations are also shown in the Figure 5 for better visualization.

## Discussion

**FIGURE 6:**
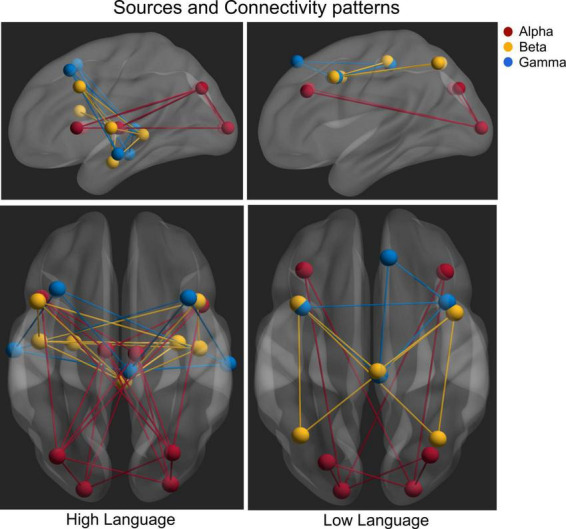
Summarized view of all the sources and connectivity highlighting the difference between high language and low language CI groups.

The current study focused on children who have CIs with disparate language and reading abilities and timing differences in hearing restoration in order to identify the underlying neural functional differences. From the analysis of resting state EEG, we identified coherent neural sources in alpha, beta and gamma frequency bands that are known to reflect different neurofunctional properties (see Figure 6 for a summarized view). The specific neural differences in sources and their connectivity in the CI groups were related to language and reading skills differences. The differences for each CI group and the potential implications related to the language and reading skills and age of hearing intervention for the two CI groups are detailed below.

### Delta and theta band differences

Research has shown that delta and theta band activity are involved in speech and language processing. Delta band activity has been previously linked with cognitive processing (Harmony, 2013), processing sentential structure of speech (Lu et al., 2022; Slaats et al., 2023), encoding higher level speech comprehension (Etard and Reichenbach, 2019), and have been shown to be altered in children with developmental language disorders (Keshavarzi et al., 2022; Gul et al., 2023). Similarly, theta band activity has also been shown to be critical for effective communication between different brain regions involved in language processing with association to better verbal working memory capacity (Wu et al., 2007; Sauseng et al., 2010; Jausovec and Jausovec, 2014), acting as an interface for language and memory (Pu et al., 2020), modulation during reading or speech listening (Rohm et al., 2001; Bastiaansen et al., 2005; Rommers et al., 2017) and allocating attention while reading from the screen (Zivan et al., 2023). In this study, we found that the CI children differed significantly with the normal hearing group in both delta and theta bands suggesting an abnormal oscillatory activity in these frequency bands. However, there were no significant differences between the high language and low language CI groups that could explain the reading and language performance differences in the standardized measures. While it appears that CI children in the current study differ in terms of source strength in these two frequency bands, the difference does not appear to contribute to the skill levels in reading and language that were measured in the current study. Subsequent studies will be needed to investigate how these differences might be related to other aspects of outcomes for CI children.

### Alpha band differences

Alpha band activity is generally associated with a range of processes from functional inhibition, cortical idling, working memory to attention modulation (Pfurtscheller et al., 1996; Klimesch, 1999, 2012; Jensen and Mazaheri, 2010; Foxe and Snyder, 2011). Overall, alpha activity plays an active role in task-dependent neuronal processing modulating visual information (Palva and Palva, 2007; Zhou et al., 2021). There were two regions exhibiting increased alpha band coherence and bidirectional connectivity for the two CI groups: the primary visual cortex and the angular gyrus, both part of the dorsal visual stream (Mishkin and Ungerleider, 1982; Goodale and Milner, 1992). The increased coherence and connectivity of sources across the two CI groups suggests that increased dorsal stream functioning is a consequence of early deafness and generally consistent with research highlighting enhanced visual processing in CI recipients relative to their NH peers (Doucet et al., 2006; Strelnikov et al., 2013; Song et al., 2015; Prince et al., 2021). However, the HL group was also implanted earlier than the LL group suggesting that earlier implantation has a more positive effect on dorsal stream function.

Additionally, for the HL group, two alpha band sources (precentral/BA 44, and the thalamus) areas associated with language functions (Johnson and Ojemann, 2000; Crosson, 2013; Klostermann et al., 2013; Bohsali et al., 2015) and the visual cortex. These sources were bidirectionally coupled and their connectivity was related to age of bilateral CI and age of hearing aid fitting. Hence, it appears that earlier intervention influences visual input to the thalamus as well as Broca’s area that have an impact on language and subsequent reading development. Moreover, the bidirectionality of the connectivity suggests more interactive processing between these important sensory and language related areas. Together these findings suggest that increased dorsal stream engagement coupled with language related interactive connectivity may be important for tuning the neural systems impacting the subsequent speech and language development.

### Beta band differences

Beta band activity is associated with communication between cortical areas as a mechanism for network integration (von Stein and Sarnthein, 2000; Betti et al., 2021) and is associated with language processing (Weiss and Mueller, 2012). Specifically, increased beta band coherence increases with the processing of visual or auditory presentations of word categories (nouns, proper names, verbs, etc.) (Weiss and Rappelsberger, 1998; Weiss and Mueller, 2003), as well as with syntactic (Bastiaansen et al., 2010) and semantic binding of lexical categories (von Stein et al., 1999; Weiss and Mueller, 2003). For the HL group, the coherent sources in the beta band were all in regions that impact sensory (thalamus), language processing (IFG, insula) and learning (hippocampus). Moreover, the connectivity of these regions was almost exclusively bidirectional. As shown from the SVM result for the HL group, the increased coherence and the communication between these sources are important for successful outcomes in developing language and reading.

In contrast, the sources from the LL group analysis were localized to different brain regions including the frontal eye fields, the SMA/posterior cingulate and the angular gyrus. The angular gyrus and frontal eye fields (FEF) are part of the dorsal attention network associated with top-down attentional processes driven by beta band activity (Engel and Fries, 2010; Miller and Buschman, 2013) suggesting that the unidirectional connectivity (angular gyrus to FEF) may be reflecting a mechanism for increasing visual attention to compensate for reduced speech recognition skill as observed in our previous study (Wolfe et al., 2021). The increased coherence and bidirectional connectivity to the FEF for the SMA/dorsal PCC suggests additional sensorimotor tuning of eye movement to maximize visual input. Overall, the beta band sources and their connectivity for the LL group suggests interactions that appear to be mainly focused on enhancing visual input.

The relationship of the sources and their connectivity to the timing of hearing intervention provides some interesting contrasts. Examination of the two beta sources for the HL group, suggest that earlier implantation is associated with more contributions from auditory processing areas (posterior insula/Heschl’s) leading to better multisensory (auditory-visual) interactions and better language and reading skill development in the longer-term. Conversely, for the later implanted group, the connectivity of the hippocampus with the thalamus and the lack of sensory representations seen in the HL group, suggests that later implantation may lead to reduced audio-visual processing negatively impacting the acquisition of age-appropriate reading skill.

### Gamma band differences

Gamma band activity is generally widely distributed and participates in various cognitive functions including perceptual binding, attention, and memory (Gray and Singer, 1989; Tallon-Baudry and Bertrand, 1999) through a network of spatially segregated brain areas (Bertrand and Tallon-Baudry, 2000). Increased coherence amplitude differed for the two CI groups with increased coherence for the HL group in the mid frontal gyrus, the middle temporal gyrus and the posterior thalamus all of which have been associated with the multiple-demand system (Duncan, 2010) and the development of literacy (Koyama et al., 2017). The middle temporal gyrus was bidirectionally connected to the mid frontal gyrus and the posterior thalamus. This network reflects a set of interactions that are optimal for binding brain areas that are critical for developing literate language.

For the LL group, gamma band connections (SFG– > PCG) were related to age of first CI and age of bilateral CI. The SFG is engaged for working memory and children with CI have been shown to perform more poorly in such tasks compared to NH children (Yee et al., 2010; Lazard et al., 2013). One possibility is that the later the intervention the more this circuit is required to engage working memory processes. The HL group showed no differences from the NH group with either of these brain areas suggesting that this increase in coherence and connectivity in the LL group maybe a potential marker for difficulty with working memory.

### Adaptative variability in neural function and successful CI outcomes

Both HL and LL group showed differences in source localization and connectivity at a network level. The differences in the two CI groups were related generally to the time at which hearing was restored clearly supporting the beneficial effect of early intervention on successful literacy-related outcomes. The differences provide also evidence for the early and later developmental effects of sensory deprivation and hearing restoration in which the cortical changes are not always accompanied by enhancements in behavioral abilities but may be associated with avoiding undesirable physiological consequences of the sensory deafferentation (Singh et al., 2018). One of the caveats related to hearing intervention, however, is that the timing of cochlear implantation for the two CI cohorts was quite variable. Hence the results related to age of intervention are to be taken with some caution. With the current recommendations for early surgical intervention by 12 months (Varadarajan et al., 2021), future cross-sectional and longitudinal studies will provide improved ability to identify factors associated with successful CI outcomes.

### Overall summary

It has been clearly established that congenital sensorineural hearing loss has an impact on neural structure and function in children (Campbell and Sharma, 2016; Xia et al., 2017; Calcus et al., 2019; Wang et al., 2019; Simon et al., 2020; Bonna et al., 2021; Deroche et al., 2023). In the present study, changes in neural organization and how they are related to subsequent development of language and reading provide insight into the neural adaptation after cochlear implantation. An important aspect of the present study is that these differences were obtained relatively task-free and coupled with a machine learning approach, where we were able to demonstrate that the differences in resting state organization for the different CI groups were related to their current language and reading skills. As such, and as shown in other studies, resting state activity plays a fundamental role in brain and behavioral function (Smith et al., 2009).

From these data there were also some clear patterns or biomarkers that are associated with the development of better language and reading in children with CIs. Increased coherence and bidirectional connectivity were associated with earlier implantation and better language and reading skill, suggesting that interactive connectivity is an important property of developing optimal processing and is important for network communication and tuning. Similarly, earlier implantation was accompanied by increased activity in speech and language related brain areas which appears to be a positive factor post implantation. In contrast, increased activity in brain areas that are secondary to speech and language development seem to be maladaptive, possibly indicating the lack of availability of some early developmental processes secondary to later hearing restoration.

### Limitations

There are few limitations of this study. As in the case for most of the studies for CI population, there is some form of heterogeneity in the data obtained. We limited this heterogeneity by having all children implanted before 4 years old, but some differences remain, four were unilaterally implanted, one participant who was bilaterally implanted was tested with a single implant during testing (processor was misplaced) and one of the 4 unilaterally implanted children wears a hearing aid (taken off during the experiment) in the non-implanted ear while 45 were bilaterally implanted. One might be tempted to consider unilaterally implanted cases as outliers, but there may not be a great difference between a bilateral user who received their second implant several years after the first, and a unilateral user. Moreover, the sort of asymmetry in EEG activity for unilateral CI implanted participants (Cartocci et al., 2018, 2019) may well occur for the former too. Beyond this, there are many aspects of surgery, etiology, neural survival, electrode-to-neuron contact, device type, processing strategy, etc., which all differ across children that could have a non-negligible impact on resting-state connectivity but unavoidable. Hence, we suggest that the current results were minimally impacted by unilateral vs. bilateral implantation, no more so than the well-known heterogeneity in this population.

Another limitation is the methodological approach undertaken in the study. As any other analytic technique, the electrical source imaging (ESI) analysis has limitations for source localization calculations. The EEG is sensitive to signal noise, which can come from background noise, distortions, and movement artifacts. These potential noise sources can affect the accuracy of the inverse solution, producing ghost sources, or even displace the predicted brain activity areas (Whittingstall et al., 2003; Zorzos et al., 2021). However, with the recent advancement in the application of sophisticated algorithms like beamforming, which is the basis for the source analyses used in the current study, some limitations have been mitigated. The algorithm operates in an iterative manner finding the first strongest coherence source and then considers that source as noise in the second iteration to find the next strongest source. Hence, this removes the possibility of the influence of one source on another. Additionally, in our earlier studies using direct LFP recordings from thalamus with a simultaneous 64-channel EEG in orthostatic tremor patients, we showed that the time frequency dynamics of the local field potentials were similar to the activity reconstructed from the beamformer extracted source signal from EEG (Muthuraman et al., 2013). Moreover, in a combined EEG and MEG study, we were able to further estimate and validate the findings from the previous study using the same spatial filter and the linear constrained minimum variance (Muthuraman et al., 2014). In the last years, researchers have used and validated this algorithm by identifying cortical and sub-cortical sources (Seeber et al., 2019; Alberto et al., 2021) and we were able to do the same with even lower spatial sampling (Muthuraman et al., 2012, 2018; Tamás et al., 2018).

One other potential limitation of the current study was that the CI group was tested with their implants/processor turned off to avoid electrical artifacts on the EEG signal. It is worth noting that temporary sound deprivation is not a rare event for a CI user which they experience on a regular basis. Hence, it is hard to imagine that resting activity created a novel state due to temporary sound deprivation. Moreover, taking an implant off is not like occluding one’s ear for a NH individual; it doesn’t cause the physiological responses like heart rate, breathing, swallowing to become enhanced. Further, the recordings occurred in quiet conditions: the background noise level in the testing room was measured at 35 dBA. Therefore, the presence or absence of low-level ambient sound would be identical for both CI groups and the difference between the CI children and the NH group would be minimal.

## Conclusion

Overall, auditory deprivation during development impacts neural organization primarily related to the processing of sensory input within specific brain regions and the interregional connectivity linking speech, language, and cognitive brain regions. In children with CIs, specific brain functional organization appears to have an impact on the development of language and reading. Some of the adaptive changes in the brain appear to be mitigated by early intervention that restore as much hearing as possible. The current results point to some of the favorable neural changes that appear to facilitate language development.

## Data availability statement

The raw data supporting the conclusions of this article will be made available by the authors, without undue reservation.

## Ethics statement

The studies involving human participants were reviewed and approved for all the experimental procedure by the Ethical Committee Board of the Institution (Hearts for Hearing) and subjects’ written informed consent was obtained from their legal guardian. All study procedures were conducted in accordance with appropriate guidelines and following the norms of the Declaration of Helsinki. Written informed consent to participate in this study was provided by the participants’ legal guardian/next of kin.

## Author contributions

NK: conceptualization, methodology, formal analysis, writing-original draft, and writing-review and editing. MD: conceptualization, investigation, data curation, and writing-review and editing. JW, SN, AB, DD, and MG: resources, data curation, and writing-review and editing. MM: software, supervision, project administration, and writing-review and editing. VG: funding acquisition, project administration, supervision, writing-original draft, and writing - review and editing. All authors contributed to the article and approved the submitted version.
